# Supplementary material for: A Rapid Immunochromatographic Method Based on Gold Nanoparticles for the Determination of Imidacloprid on Fruits and Vegetables
Source: Foods. 2023 Jan 23;12(3):512. doi: 10.3390/foods12030512 (PMC9914284; doi:10.3390/foods12030512)
Supplement: Supplementary file 1 [file foods-12-00512-s001.zip › foods-2140881-supplementary.pdf]

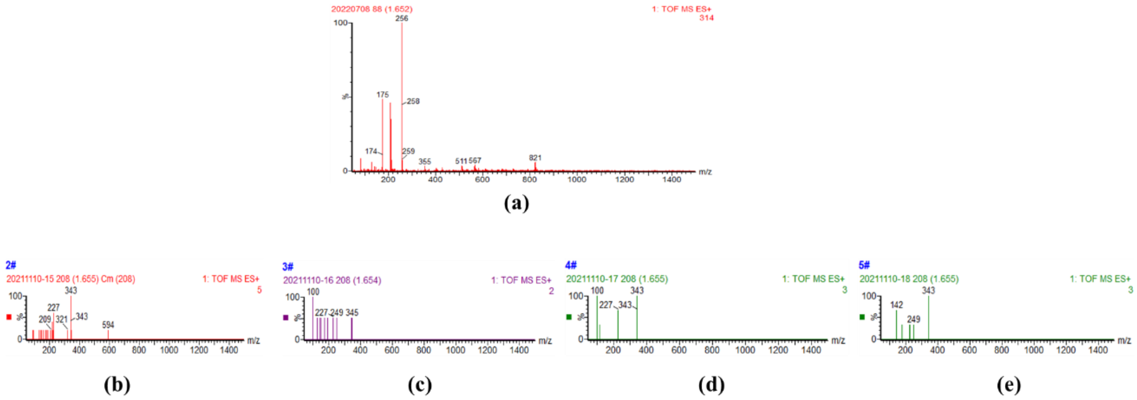

Figure. S1. LC-MS analysis of (a) IMP standard (1000 ng mL^{-1}); negative sample of Chinese cabbage (b); cowpea (c); apple (d); and pear (e).
